# (*E*)-3-(1-Methyl-1*H*-pyrrol-2-yl)-1-phenyl­prop-2-en-1-one

**DOI:** 10.1107/S1600536811009214

**Published:** 2011-03-15

**Authors:** Li Liu, Jian Li, Ying Shao

**Affiliations:** a Analytical Center, Changzhou University, Changzhou 213164, People’s Republic of China; b School of Pharmaceutical Engineering and Life Science, Changzhou University, Changzhou 213164, People’s Republic of China; c Key Laboratory of Fine Petrochemical Technology, Changzhou University, Changzhou 213164, People’s Republic of China

## Abstract

The crystal structure of the title compound, C_14_H_13_NO, exhibits an *E* configuration. The conjugated compound is slightly twisted with a dihedral angle of 29.3° between the benzene and pyrrole rings. Two inter­molecular C—H⋯O inter­actions lead to a dimer. In the crystal, intermolecular C—H⋯O interactions generate an inversion dimer.

## Related literature

For related literature on chalcone and its derivatives, see: Kelly *et al.* (2004 ▶); Takahashi *et al.* (2005 ▶). For the anti­cancer properties of chalcone derivatives, see: Zi & Simoneau (2005 ▶); Bennasroune *et al.* (2004 ▶); Moriarty *et al.* (2006 ▶). For a related structure, see Jing (2009 ▶).
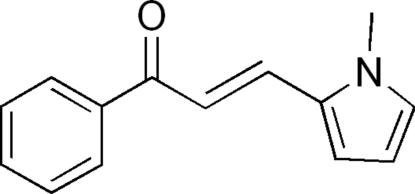


## Experimental

### 

#### Crystal data

C_14_H_13_NO
*M* *_r_* = 211.25
Monoclinic, 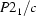

*a* = 13.209 (2) Å
*b* = 4.8849 (9) Å
*c* = 18.036 (3) Å
β = 102.394 (4)°
*V* = 1136.6 (4) Å^3^
*Z* = 4
Mo *K*α radiation
μ = 0.08 mm^−1^
*T* = 296 K
0.25 × 0.22 × 0.20 mm

#### Data collection

Bruker APEX CCD diffractometer
Absorption correction: multi-scan (*SADABS*; Bruker, 2000 ▶) *T*_min_ = 0.981, *T*_max_ = 0.985
5920 measured reflections
1996 independent reflections
1459 reflections with *I* > 2σ(*I*)
*R*_int_ = 0.036

#### Refinement

*R*[*F* ^2^ > 2σ(*F* ^2^)] = 0.043
*wR*(*F* ^2^) = 0.153
*S* = 1.00
1996 reflections
146 parameters
1 restraint
H-atom parameters constrained
Δρ_max_ = 0.12 e Å^−3^
Δρ_min_ = −0.19 e Å^−3^

### 

Data collection: *SMART* (Bruker, 2000 ▶); cell refinement: *SAINT* (Bruker, 2000 ▶); data reduction: *SAINT*; program(s) used to solve structure: *SHELXS97* (Sheldrick, 2008 ▶); program(s) used to refine structure: *SHELXL97* (Sheldrick, 2008 ▶); molecular graphics: *SHELXTL* (Sheldrick, 2008 ▶); software used to prepare material for publication: *SHELXTL*.

## Supplementary Material

Crystal structure: contains datablocks I, global. DOI: 10.1107/S1600536811009214/fl2335sup1.cif

Structure factors: contains datablocks I. DOI: 10.1107/S1600536811009214/fl2335Isup2.hkl

Additional supplementary materials:  crystallographic information; 3D view; checkCIF report

## Figures and Tables

**Table 1:** Hydrogen-bond geometry (Å, °)

*D*—H⋯*A*	*D*—H	H⋯*A*	*D*⋯*A*	*D*—H⋯*A*
C1—H1*C*⋯O1^i^	0.96	2.49	3.434 (2)	169

Symmetry code: (i) 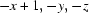.

**Fig. 1.**
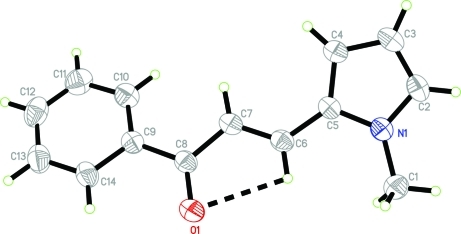
The molecular structure of the title compound drawn with 30% probability ellipsoids.

**Fig. 2.**
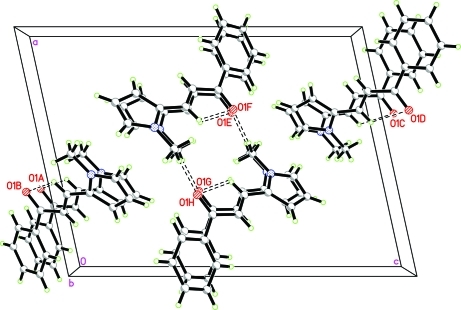
Packing diagram of title compound showing the hydrogen-bonding dimer.

## supplementary crystallographic information

### Comment

Chalcone and its derivatives have been of interest because they can serve as precursors for the biosynthesis of flavonoids and substrates for the evaluation of many organic reactions (Kelly *et al.*, 2004; Takahashi *et al.*, 2005). The most important naturally occurring chalcone has shown potential as a drug candidate, flavokawain A from kava extracts which has strong anti-proliferative and apoptotic effects against human bladder cancer cells (Zi *et al.*, 2005). Pyrrole-based derivatives were also reported as potent anticancer agents (Bennasroune *et al.*, 2004; Moriarty *et al.*, 2006). We now report the structure of a chalcone derivative with an *N*-methyl pyrrole group.

The title compound exists as the most stable (*E)-*configuration (Fig.1). The pyrrole ring is connected to the phenyl group through the C5—C6=C7—C8—C9 chain with the C=C bond length being 1.332 (3) Å. The dihedral angle between the benzene ring and pyrrole ring is 29.3°, larger than that of (*E*)-3-(4-Fluorophenyl)-1-phenyl-2- propen-1-one (Jing, 2009) which demonstrate that the pyrrole unit influences the twist between the two rings.

There is a intramolecular C6—H6···O1 interaction between the carbonyl and olefinic H atom (Table 1). In its packing structure, hydrogen-bonded dimers are formed *via* intermolecular C1—H1C···O1 interactionss (Fig.2).

### Experimental

A solution of 1-methylpyrrole-2-carboxaldehyde (0.20 g, 1.8 mmol) in ethanol (15 ml) was added slowly to a mixture of acetophenone (0.22 g, 1.8 mmol) and KOH (0.10 g, 1.8 mmol) in methanol (30 ml) over 30 minutes at room temperature. The mixture was stirred for 16 h, and the yellow solid (0.31 g, 88.6%) was collected by filtration. Single crystals suitable for X-ray diffraction were obtained after recrystallization from ethanol.

### Refinement

Methyl H atoms were placed in calculated positions, with C—H = 0.96 Å, and refined using a riding model, with *U*iso(H) = 1.5*U*eq(C). Benzene and ethylene H atoms were also assigned to calculated positions with C—H = 0.93 Å, and refined using a riding model, with *U*iso(H) = 1.2*U*eq(C).

### Crystal data

**Table:**

C_14_H_13_NO	*F*(000) = 448
*M**_r_* = 211.25	*D*_x_ = 1.235 Mg m^−^^3^
Monoclinic, *P*2_1_/*c*	Mo *K*α radiation, λ = 0.71073 Å
Hall symbol: -P 2ybc	Cell parameters from 1712 reflections
*a* = 13.209 (2) Å	θ = 2.3–27.0°
*b* = 4.8849 (9) Å	µ = 0.08 mm^−^^1^
*c* = 18.036 (3) Å	*T* = 296 K
β = 102.394 (4)°	Prism, yellow
*V* = 1136.6 (4) Å^3^	0.25 × 0.22 × 0.20 mm
*Z* = 4	

### Data collection

**Table:**

Bruker APEXII CCD diffractometer	1996 independent reflections
Radiation source: fine-focus sealed tube	1459 reflections with *I* > 2σ(*I*)
graphite	*R*_int_ = 0.036
φ and ω scans	θ_max_ = 25.0°, θ_min_ = 1.6°
Absorption correction: multi-scan (*SADABS*; Bruker, 2000)	*h* = −14→15
*T*_min_ = 0.981, *T*_max_ = 0.985	*k* = −5→5
5920 measured reflections	*l* = −21→17

### Refinement

**Table:**

Refinement on *F*^2^	Primary atom site location: structure-invariant direct methods
Least-squares matrix: full	Secondary atom site location: difference Fourier map
*R*[*F*^2^ > 2σ(*F*^2^)] = 0.043	Hydrogen site location: inferred from neighbouring sites
*wR*(*F*^2^) = 0.153	H-atom parameters constrained
*S* = 1.00	*w* = 1/[σ^2^(*F*_o_^2^) + (0.1*P*)^2^ + 0.038*P*] where *P* = (*F*_o_^2^ + 2*F*_c_^2^)/3
1996 reflections	(Δ/σ)_max_ < 0.001
146 parameters	Δρ_max_ = 0.12 e Å^−^^3^
1 restraint	Δρ_min_ = −0.19 e Å^−^^3^

### Special details

**Table:**

Geometry. All e.s.d.'s (except the e.s.d. in the dihedral angle between two l.s. planes) are estimated using the full covariance matrix. The cell e.s.d.'s are taken into account individually in the estimation of e.s.d.'s in distances, angles and torsion angles; correlations between e.s.d.'s in cell parameters are only used when they are defined by crystal symmetry. An approximate (isotropic) treatment of cell e.s.d.'s is used for estimating e.s.d.'s involving l.s. planes.
Refinement. Refinement of *F*^2^ against ALL reflections. The weighted *R*-factor *wR* and goodness of fit *S* are based on *F*^2^, conventional *R*-factors *R* are based on *F*, with *F* set to zero for negative *F*^2^. The threshold expression of *F*^2^ > σ(*F*^2^) is used only for calculating *R*-factors(gt) *etc*. and is not relevant to the choice of reflections for refinement. *R*-factors based on *F*^2^ are statistically about twice as large as those based on *F*, and *R*- factors based on ALL data will be even larger.

### Fractional atomic coordinates and isotropic or equivalent isotropic displacement parameters (Å^2^)

**Table:**

	*x*	*y*	*z*	*U*_iso_*/*U*_eq_	
C1	0.49092 (14)	0.6200 (4)	0.10561 (11)	0.0666 (5)	
H1A	0.5321	0.7628	0.1338	0.100*	
H1B	0.4586	0.6867	0.0561	0.100*	
H1C	0.5345	0.4668	0.1006	0.100*	
C2	0.39810 (16)	0.6409 (4)	0.21180 (10)	0.0628 (5)	
H2	0.4367	0.7835	0.2380	0.075*	
C3	0.31956 (16)	0.5074 (4)	0.23440 (11)	0.0671 (5)	
H3	0.2954	0.5409	0.2783	0.080*	
C4	0.28227 (14)	0.3116 (4)	0.17956 (10)	0.0599 (5)	
H4	0.2286	0.1892	0.1804	0.072*	
C5	0.33895 (13)	0.3300 (3)	0.12319 (9)	0.0497 (4)	
C6	0.33082 (14)	0.1826 (3)	0.05417 (9)	0.0528 (5)	
H6	0.3806	0.2200	0.0261	0.063*	
C7	0.25918 (14)	−0.0039 (4)	0.02554 (10)	0.0555 (5)	
H7	0.2072	−0.0442	0.0514	0.067*	
C8	0.26049 (14)	−0.1460 (3)	−0.04539 (9)	0.0533 (5)	
C9	0.16992 (13)	−0.3136 (3)	−0.08205 (9)	0.0522 (5)	
C10	0.07359 (15)	−0.2885 (4)	−0.06423 (11)	0.0693 (6)	
H10	0.0645	−0.1675	−0.0264	0.083*	
C11	−0.00986 (17)	−0.4435 (5)	−0.10267 (13)	0.0820 (6)	
H11	−0.0748	−0.4233	−0.0911	0.098*	
C12	0.00353 (19)	−0.6241 (5)	−0.15709 (13)	0.0828 (7)	
H12	−0.0520	−0.7295	−0.1820	0.099*	
C13	0.0988 (2)	−0.6518 (4)	−0.17544 (13)	0.0791 (6)	
H13	0.1074	−0.7752	−0.2128	0.095*	
C14	0.18079 (16)	−0.4978 (4)	−0.13874 (10)	0.0636 (5)	
H14	0.2448	−0.5164	−0.1518	0.076*	
N1	0.41137 (10)	0.5347 (3)	0.14553 (7)	0.0526 (4)	
O1	0.33614 (10)	−0.1304 (3)	−0.07478 (7)	0.0721 (4)	

### Atomic displacement parameters (Å^2^)

**Table:**

	*U*^11^	*U*^22^	*U*^33^	*U*^12^	*U*^13^	*U*^23^
C1	0.0590 (11)	0.0738 (12)	0.0670 (12)	−0.0017 (10)	0.0131 (9)	−0.0013 (10)
C2	0.0711 (12)	0.0597 (11)	0.0545 (10)	0.0080 (9)	0.0067 (9)	−0.0111 (9)
C3	0.0788 (13)	0.0739 (12)	0.0514 (11)	0.0111 (10)	0.0204 (9)	−0.0058 (9)
C4	0.0664 (11)	0.0655 (11)	0.0496 (10)	0.0013 (9)	0.0164 (8)	0.0008 (8)
C5	0.0557 (10)	0.0477 (9)	0.0449 (9)	0.0075 (8)	0.0091 (7)	0.0017 (7)
C6	0.0621 (10)	0.0502 (9)	0.0477 (9)	0.0061 (8)	0.0152 (8)	0.0039 (7)
C7	0.0610 (11)	0.0597 (10)	0.0471 (9)	0.0040 (8)	0.0149 (8)	0.0000 (8)
C8	0.0607 (10)	0.0529 (10)	0.0468 (9)	0.0049 (8)	0.0126 (8)	0.0016 (7)
C9	0.0605 (11)	0.0487 (9)	0.0460 (9)	0.0056 (8)	0.0082 (7)	0.0069 (7)
C10	0.0669 (12)	0.0757 (12)	0.0644 (12)	0.0061 (10)	0.0120 (9)	−0.0022 (10)
C11	0.0627 (13)	0.0969 (15)	0.0819 (15)	−0.0026 (12)	0.0056 (11)	0.0084 (13)
C12	0.0885 (16)	0.0747 (14)	0.0731 (14)	−0.0180 (12)	−0.0095 (12)	0.0096 (11)
C13	0.0952 (16)	0.0692 (13)	0.0691 (13)	−0.0103 (12)	0.0090 (12)	−0.0103 (10)
C14	0.0760 (13)	0.0622 (11)	0.0505 (10)	0.0003 (9)	0.0085 (9)	−0.0053 (8)
N1	0.0548 (9)	0.0533 (8)	0.0483 (8)	0.0064 (7)	0.0081 (6)	0.0003 (6)
O1	0.0733 (9)	0.0885 (10)	0.0591 (8)	−0.0093 (7)	0.0244 (7)	−0.0155 (7)

### Geometric parameters (Å, °)

**Table:**

C1—N1	1.456 (2)	C7—C8	1.459 (2)
C1—H1A	0.9600	C7—H7	0.9300
C1—H1B	0.9600	C8—O1	1.2298 (19)
C1—H1C	0.9600	C8—C9	1.483 (2)
C2—N1	1.349 (2)	C9—C10	1.383 (2)
C2—C3	1.360 (3)	C9—C14	1.392 (2)
C2—H2	0.9300	C10—C11	1.393 (3)
C3—C4	1.388 (3)	C10—H10	0.9300
C3—H3	0.9300	C11—C12	1.359 (3)
C4—C5	1.389 (2)	C11—H11	0.9300
C4—H4	0.9300	C12—C13	1.375 (3)
C5—N1	1.383 (2)	C12—H12	0.9300
C5—C6	1.422 (2)	C13—C14	1.367 (3)
C6—C7	1.335 (2)	C13—H13	0.9300
C6—H6	0.9300	C14—H14	0.9300
			
N1—C1—H1A	109.5	O1—C8—C7	120.81 (16)
N1—C1—H1B	109.5	O1—C8—C9	119.64 (15)
H1A—C1—H1B	109.5	C7—C8—C9	119.56 (16)
N1—C1—H1C	109.5	C10—C9—C14	118.22 (17)
H1A—C1—H1C	109.5	C10—C9—C8	122.82 (16)
H1B—C1—H1C	109.5	C14—C9—C8	118.92 (16)
N1—C2—C3	109.48 (17)	C9—C10—C11	120.35 (19)
N1—C2—H2	125.3	C9—C10—H10	119.8
C3—C2—H2	125.3	C11—C10—H10	119.8
C2—C3—C4	107.09 (17)	C12—C11—C10	120.0 (2)
C2—C3—H3	126.5	C12—C11—H11	120.0
C4—C3—H3	126.5	C10—C11—H11	120.0
C3—C4—C5	108.21 (16)	C11—C12—C13	120.4 (2)
C3—C4—H4	125.9	C11—C12—H12	119.8
C5—C4—H4	125.9	C13—C12—H12	119.8
N1—C5—C4	106.34 (14)	C14—C13—C12	120.0 (2)
N1—C5—C6	122.59 (15)	C14—C13—H13	120.0
C4—C5—C6	131.06 (16)	C12—C13—H13	120.0
C7—C6—C5	126.73 (17)	C13—C14—C9	121.0 (2)
C7—C6—H6	116.6	C13—C14—H14	119.5
C5—C6—H6	116.6	C9—C14—H14	119.5
C6—C7—C8	121.53 (17)	C2—N1—C5	108.86 (15)
C6—C7—H7	119.2	C2—N1—C1	124.94 (16)
C8—C7—H7	119.2	C5—N1—C1	126.19 (14)
			
N1—C2—C3—C4	−0.4 (2)	C8—C9—C10—C11	177.39 (17)
C2—C3—C4—C5	−0.4 (2)	C9—C10—C11—C12	1.2 (3)
C3—C4—C5—N1	1.03 (18)	C10—C11—C12—C13	−1.1 (3)
C3—C4—C5—C6	−178.39 (17)	C11—C12—C13—C14	0.2 (3)
N1—C5—C6—C7	−175.01 (15)	C12—C13—C14—C9	0.6 (3)
C4—C5—C6—C7	4.3 (3)	C10—C9—C14—C13	−0.6 (3)
C5—C6—C7—C8	−178.64 (15)	C8—C9—C14—C13	−178.38 (16)
C6—C7—C8—O1	11.5 (3)	C3—C2—N1—C5	1.1 (2)
C6—C7—C8—C9	−169.16 (15)	C3—C2—N1—C1	−177.72 (16)
O1—C8—C9—C10	−163.32 (17)	C4—C5—N1—C2	−1.29 (18)
C7—C8—C9—C10	17.3 (2)	C6—C5—N1—C2	178.19 (15)
O1—C8—C9—C14	14.4 (2)	C4—C5—N1—C1	177.48 (15)
C7—C8—C9—C14	−164.97 (15)	C6—C5—N1—C1	−3.0 (2)
C14—C9—C10—C11	−0.3 (3)		

### Hydrogen-bond geometry (Å, °)

**Table:**

*D*—H···*A*	*D*—H	H···*A*	*D*···*A*	*D*—H···*A*
C1—H1C···O1^i^	0.96	2.49	3.434 (2)	169
C6—H6···O1	0.93	2.48	2.797 (2)	100

Symmetry codes: (i) −*x*+1, −*y*, −*z*.
